# Substitution at rt269 in Hepatitis B Virus Polymerase Is a Compensatory Mutation Associated with Multi-Drug Resistance

**DOI:** 10.1371/journal.pone.0136728

**Published:** 2015-08-31

**Authors:** Sung Hyun Ahn, Doo Hyun Kim, Ah Ram Lee, Beom Kyung Kim, Yong Kwang Park, Eun-Sook Park, Sang Hoon Ahn, Gu-Choul Shin, Soree Park, Hong Seok Kang, Jin-Kyu Rhee, Sung-Il Yang, Youhoon Chong, Kyun-Hwan Kim

**Affiliations:** 1 Department of Pharmacology, Center for Cancer Research and Diagnostic Medicine, IBST, School of Medicine, Konkuk University, Seoul, Korea; 2 Department of Internal Medicine and Institute of Gastroenterology, Yonsei University College of Medicine, Seoul, Korea; 3 KU Open Innovation Center, Konkuk University, Seoul, Korea; 4 Department of Bioscience and Biotechnology, Bio/Molecular Informatics Center, Konkuk University, Seoul, Korea; 5 Departments of Food Science and Engineering, Ewha Womans University, Seoul, Korea; 6 Research Institute of Medical Sciences, Konkuk University, Seoul, Korea; University of Cincinnati College of Medicine, UNITED STATES

## Abstract

The emergence of compensatory mutations in the polymerase gene of drug resistant hepatitis B virus (HBV) is associated with treatment failure. We previously identified a multi-drug resistant HBV mutant, which displayed resistance towards lamivudine (LMV), clevudine (CLV), and entecavir (ETV), along with a strong replication capacity. The aim of this study was to identify the previously unknown compensatory mutations, and to determine the clinical relevance of this mutation during antiviral therapy. *In vitro* mutagenesis, drug susceptibility assay, and molecular modeling studies were performed. The rtL269I substitution conferred 2- to 7-fold higher replication capacity in the wild-type (WT) or YMDD mutation backbone, regardless of drug treatment. The rtL269I substitution alone did not confer resistance to LMV, ETV, adefovir (ADV), or tenofovir (TDF). However, upon combination with YMDD mutation, the replication capacity under LMV or ETV treatment was enhanced by several folds. Molecular modeling studies suggested that the rtL269I substitution affects template binding, which may eventually lead to the enhanced activity of rtI269-HBV polymerase in both WT virus and YMDD mutant. The clinical relevance of the rtL269I substitution was validated by its emergence in association with YMDD mutation in chronic hepatitis B (CHB) patients with sub-optimal response or treatment failure to LMV or CLV. Our study suggests that substitution at rt269 in HBV polymerase is associated with multi-drug resistance, which may serve as a novel compensatory mutation for replication-defective multi-drug resistant HBV.

## Introduction

Long-term outcomes of chronic hepatitis B virus (HBV) infection, including inflammation, cirrhosis, and hepatocellular carcinoma (HCC), are major medical problems worldwide [1]. World Health Organization (WHO) estimates approximately 350 to 400 million people to be afflicted with chronic hepatitis B (CHB) infections [1]. Antiviral treatment for CHB ameliorates liver disease and prevents disease progression to HCC. Although several oral antiviral agents have been introduced for the treatment of CHB over the last two decades, a long-term antiviral therapy is required for majority of patients. However, such a long-term therapy could cause the emergence of drug resistance [2].

Currently approved, nucleos(t)ide analogues (NAs) to treat patients with CHB include lamivudine (LMV), telbivudine (LdT), adefovir (ADV), entecavir (ETV), clevudine (CLV), and tenofovir (TDF) [2–5]. Since all these clinically available HBV drugs share a common target for the viral reverse transcriptase (RT), resistance to all NAs are reported to result from specific mutations in the RT domain [2, 6–8].

LMV, the first approved anti-HBV agent, is a strong inhibitor of HBV replication. However, the resistance rate of LMV was reported to extend to 23% and 80% of HBV carriers, after one and five year(s) of monotherapy, respectively [2, 9]. The primary mutation conferring LMV resistance is rtM204I/V in the YMDD motif. Since the YMDD mutant is almost replication-defective [10], this mutation is usually accompanied by secondary (compensatory) mutations restoring genome replication. The well-characterized compensatory mutations include rtL180M, rtL80I/V, and rtV173L; they enhance replication of the rtM204I/V mutants [2, 7, 11]. Recently, substitutions at rtS117 and rtL229 have been identified as novel compensatory mutations for the YMDD mutant during lamivudine therapy [12, 13].

ETV is the most potent among the currently available anti-HBV drugs, with a very low resistance rate [2, 11, 14]. The mutations associated with ETV resistance are complex. They include rtI169T, rtL180M, rtS184S/A/I/L/G/C/M, M204I/V, rtS202G/I, and rtM250I/V. Which of these mutations confer ETV resistance and which are compensatory mutations have not yet been fully established.

CLV, a fluorinated LdT, expressed a similar resistance profile to LMV and LdT. The primary resistance mutation to CLV was also mapped to the YMDD motif. The most common mutation during viral breakthrough in CLV-failure patients is rtM204I. On the other hand, rtL229V behaved as a compensatory mutation to rescue replication of the rtM204I mutant [15].

Genotypic analyses have revealed that rtN236T and/or rtA181T/V mutations confer ADV resistance. An *in vitro* drug susceptibility assay demonstrated the association of similar mutations, namely rtA194T, rtL181T/V, and/or rtN236T, with TDF resistance [2]. However, such mutations have not yet been identified in clinical studies.

We have previously identified a multi-drug resistant HBV mutant (clone 50–2), which harbored the quintuple mutations (rtM129L+V173L+M204I+L269I+H337N) that conferred robust replicative ability and strong cross-resistance to LMV, CLV, and ETV [15]. Since the emergence of compensatory mutations in the polymerase gene of drug-resistant HBV is associated with treatment failure, the identification of these mutations might have a significant impact on the development of treatment strategies for patients with CHB.

In this study, the effect of each of the five mutations on the replication ability and drug resistance was investigated using site-directed mutagenesis and drug susceptibility assay. We discovered that substitution at rtL269 is associated with robust replication of the multi-drug resistant HBV. In addition, a molecular modeling study suggested that the rtL269I substitution affects replication. Finally, the clinical relevance of the rtL269I substitution was investigated.

## Material and Methods

### Construction of the HBV RT mutant replicons by site-directed mutagenesis

HBV isolates with WT RT domain or the rtM129L+V173L+M204I+L269I+H337N mutations were derived from patient sera (obtained from tissue bank) and converted to 1.2mer replicon, using the pGEM-4z vector (Promega Corporation, Madison, WI, USA), as previously described [15]. Other mutant clones including rtL269I, rtM204I, rtM204I+L269I, rtV173L+M204I, rtV173L+M204I+L269I, rtM129L+V173L+M204I, and rtM129L+V173L+M204I+H337N were generated from the WT HBV 1.2mer by site-directed mutagenesis [16]. Briefly, HBV 1.2mer replicons were generated by amplification of the RT domain of patient-derived HBV genome using following primers: For 5'-AAT CTT CTC GAG GAC TGG GGA CCC TGC ACC-3' (XhoI site is underlined); Rev 5'-GAG CAG CCA TGG GAA GGA GGT GTA TTT CCG-3' (NcoI site is underlined). To generate the site-directed mutagenesis in 1.2mer replicons, the specific regions of the HBV 1.2mer were amplified by overlapping extension PCR using following primers: for rtM129L, For 5'-GCA CGG GAC CCT GCA AGA CCT GC-3' and Rev 5'-GCA GGT CTT GCA GGG TCC CGT GC-3'; for rtV173L, For 5'-ATT CCT ATG GGA CTG GGC CTC AGT CCG-3' and Rev 5'-CGG ACT GAG GCC CAG TCC CAT AGG AAT-3'; for rtM204I, For 5'-TGG CTT TCA GTT ATA TCG ATG ATG TGG TAT-3' and Rev 5'-ATA CCA CAT CAT CGA TAT AAC TGA AAG CCA-3'; for rtL269I, For 5'-AAC ATA TTG TAC AAA AAA TCA AGC AAT GTT TTC G-3' and Rev 5'-CGA AAA CAT TGC TTG ATT TTT TGT ACA ATA TGT T-3'; for rtH337N, For 5'-TAA ACA ATA TCT GAA CCT TTA CCC CGT TG-3' and Rev 5'-CAA CGG GGT AAA GGT TCA GAT ATT GTT TA-3'. The PCR conditions were 95°C for 5 min, followed by 32 cycles of 95°C for 50 s, 62°C for 50 s, and 72°C for 1 min 20 s, with a final extension at 72°C for 10 min. The purified products were digested with XhoI and NcoI, and RT domain of the HB 1.2mer was swapped for the site-directed mutant. All mutant clones were verified by sequencing.

### Cell culture, transfection, and drug treatment

Huh7 human hepatoma cells were obtained from the American Type Culture Collection (Manassas, VA, USA) and cultured in Dulbecco’s modified Eagle’s medium (Gibco, Grand Island, NY, USA) supplemented with 10% fetal bovine serum (Gibco) and 1% penicillin-streptomycin (Gibco), in a 5% CO_2_ environment at 37°C. These cells were transiently transfected with 2 μg of the HBV WT and mutant 1.2mer replicons in six-well plates, using Lipofectamine 2000 (Invitrogen, Carlsbad, CA, USA).

LMV, CLV, and ETV were obtained from GlaxoSmithKline (Brentford, United Kingdom), Bukwang Phamaceutical Co. (Seoul, South Korea), and Moravek (Brea, CA, USA), respectively. ADV and TDF were obtained from Gilead Science (Foster City, CA, USA). All drugs were treated for 3 days with daily change with fresh medium containing each drug. The final treatment concentrations of the drugs were 20 μM for LMV, CLV, ADV, and TDF, and 1 μM for ETV [15].

### Drug susceptibility and replication assay

The replicative HBV DNA was extracted from intracellular core particles for determination of drug susceptibility, and analyzed by Southern blot analysis as described in previous studies [11, 17]. In brief, cells were transfected with HBV replicons, and treated with the indicated drugs for 4 days. Cells were lysed with HEPES buffer (100μl) containing 1% NP-40. The cell lysate was treated with DNase I (Clontech/Takara Bio, Mountain View, CA, USA) and mung bean nuclease (Clontech/Takara Bio) for 15 min at 37°C to remove the transfected plasmid DNA. The intracellular core particles were precipitated using 26% poly-ethylene glycol (PEG) solution and the HBV DNA was obtained by digestion of the capsid protein with proteinase K (20mg/ml, Roche Applied Science, Indianapolis, IN, USA) at 37°C for 2 h. Following the extraction of HBV DNA by phenol/chloroform and ethanol/sodium acetate precipitation, the total DNA was separated on an 0.8% agarose gel, and transferred onto a Hybond-N+ membrane (GE Healthcare, Buckinghamshire, UK). HBV DNA was detected using highly pure and randomly primed probes labeled with [α-^32^P]dCTP (500 μCi/μmol; Perkin-Elmer Inc., Waltham, MA, USA). In order to quantify the replication level, the membranes were analyzed using a Phosphorimager and Multi-Gauge v3.2 software (Fujifilm, Tokyo, Japan).

### Extracellular HBV DNA analysis

The extracellular secreted viral particles were obtained from culture medium at 3 days post-transfection. The culture supernatant was prespun at 4,000 rpm for 10 min and loaded on top of 20% sucrose in TEN buffer and ultracentrifugated at 39,000 rpm for 18 h to separate extracellular viral particles. Obtained HBV DNA was subjected to Southern blot analysis as described above.

### HBeAg measurement

The secreted HBeAg was measured to determine the transfection yield of HBV replicons. The HBeAg in culture medium was analyzed using HBeAg ELISA kit purchased from Wantai Pharm Inc. (Beijing, China). The culture supernatant was diluted tenfold, and subjected to ELISA analysis according to the manufacturer’s instruction.

### Molecular modeling

A comparative model of the HBV polymerase-DNA-TTP complex was constructed using the Prime module in the Maestro software (Maestro, version 7.5, Schrödinger, LLC (2006), New York, NY, USA), based on the sequence alignment between HBV polymerase and HIV-1 RT [18], and the crystal structure of the HIV-1 RT-DNA-dNTP ternary complex (Protein Data Bank accession no. 1RTD) [19]. An additional HBV polymerase model was constructed by rtL269I substitution. The two Mg^2+^ ions, thymidine triphosphate, and the template-primer duplex were adopted from the HIV-1 RT-DNA-dNTP complex structure (1RTD), and located at the same position in the modeled HBV polymerase structure.

The modeled HBV polymerase-DNA-TTP complex was equilibrated by energy minimizations and simulation of the molecular dynamics. Modeling studies were conducted using the MacroModel (Maestro, version 7.5; Schrödinger, LLC), which was run on the Linux platform. The complex was minimized using the OPLS_2005 force field, in the presence of the GB/SA continuum water model, until no significant movements were observed in the atomic coordinates, before performing molecular dynamic simulations. A conjugate gradient, Polak-Ribiere 1st derivative method, was used for energy minimization. Molecular dynamic simulations on the HBV polymerase-DNA-TTP complex was performed using OPLS_2005, in the presence of a GB/SA continuum water model, by heating from 0 to 300 K within 5 ps, and equilibrating at 300 K for an additional 10 ps. The production dynamic simulations were conducted for 500 ps, with a step size of 1.5 fs at 300 K. A shake algorithm was used to constrain the covalent bonds to the hydrogen atoms.

### Patients and genotyping of HBV RT genes

Chronic HBV carriers admitted to the Severance Hospital (Yonsei University Medical Center) in Seoul, Korea, were included in the study. The study was approved by the Severance Hospital Institutional Review Board, and written informed consent was obtained from participants. The four CLV-resistant patients were reported to characterize the CLV-resistant HBV, in our previous study [15]. Serological markers for HBsAg and HBeAg were determined by enzyme-linked immunoassays (Behringwerke, Marburg, Germany). The HBV DNA levels of all patients were determined by PCR, using the Roche COBAS Amplicor system (Roche Applied Diagnostics). All serum samples were obtained from the hospital tissue/blood bank and approved by the Institutional Review Board (IRB No. 4-2009-0725).

HBV DNA was extracted from the serum samples using the DNA Blood Mini Kit (Qiagen, Venlo, Netherlands) according to the manufacturer protocols. The HBV RT gene was amplified by PCR, and mutations were determined by direct sequencing [15].

### Statistical analyses

Statistical analyses were performed using the Student’s *t*-test. The P values of <0.05 were considered statistically significant.

## Results

### A multi-drug resistant HBV mutant from a CHB patient has two-fold higher replication capacity than a WT clone

**Fig 1:**
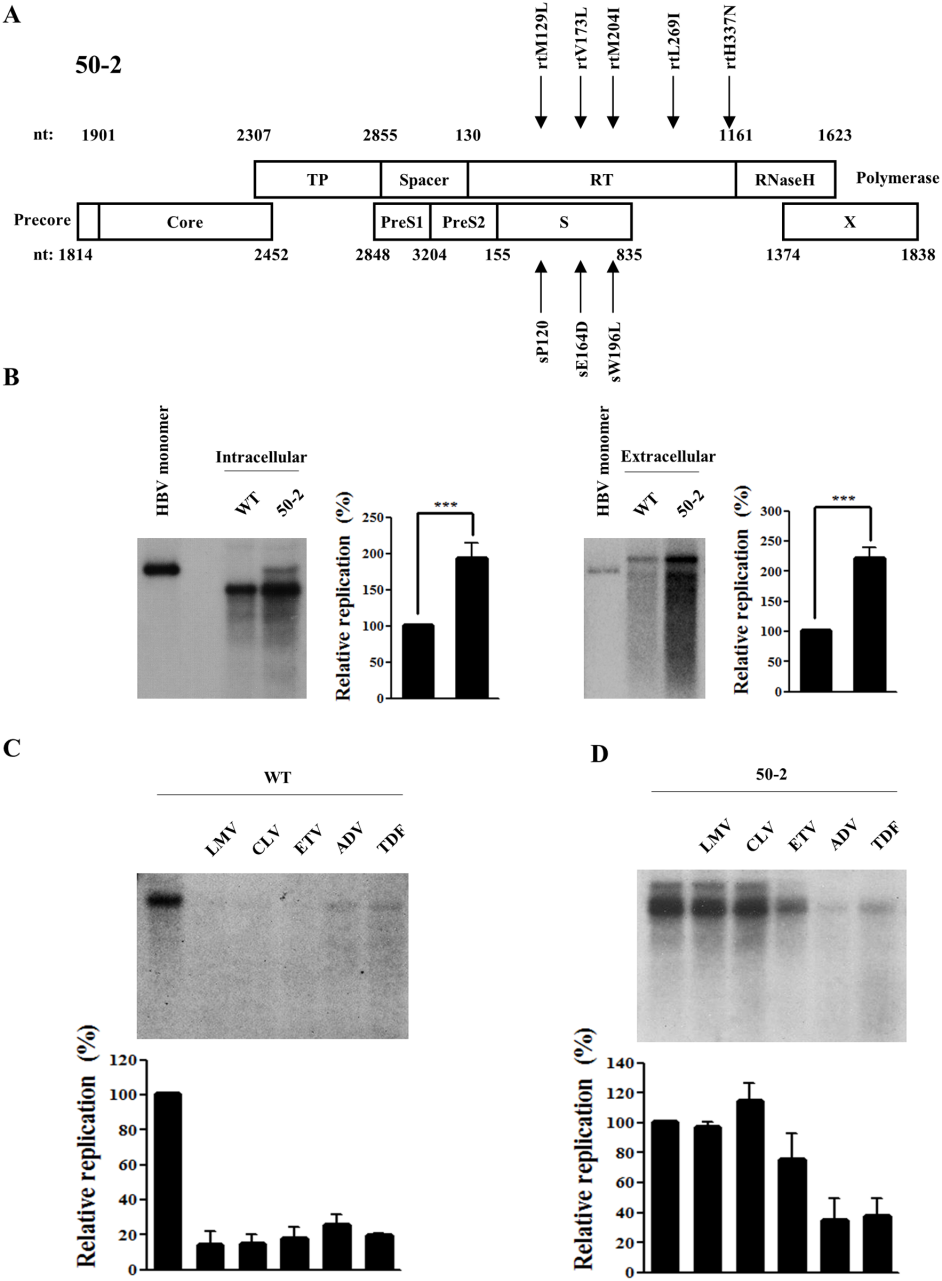
Characterization of multi-drug resistant HBV mutant 50–2 isolated from a chronic HBV patient. (A) Schematic representation of the mutations in RT and overlapping surface gene of clone 50–2. The amino acid changes in polymerase and corresponding surface gene are indicated by arrows. (B) Comparison of intracellular and extracellular secreted HBV DNA levels between WT virus and clone 50–2. The HBV 1.2mer construct plasmids (2 μg) were transfected into Huh7 hepatoma cells and harvested at 72h post-transfection. The linearized 3.2kb HBV genome was loaded in lane 1 as a marker. (C) & (D) *In vitro* susceptibility WT HBV (C) and clone 50–2 (D) to lamivudine (LMV), clevudine (CLV), entecavir (ETV), adefovir (ADV), and tenofovir (TDF). Cells were treated for 3 days with each drug, and the replication level was compared with that of the WT (without drug treatment). The relative HBV replication level was quantified using Phosphorimager. The standard deviation of three independent experiments was measured (***, *P* < 0.001).

We have previously reported a multi-drug resistant HBV mutant (clone 50–2), which harbored the quintuple rtM129L+V173L+M204I+L269I+H337N mutations in the RT domain [15]. Due to the overlap of the polymerase gene with the surface gene, these mutations are accompanied by two mutations in the surface gene; sE164D and sW196L (Fig 1A). The average half-maximal inhibitory concentration (IC_50_) values of clone 50–2 were determined to be >100, >100, 0.7, 3.5, and 5.9 μM for LMV, CLV, ETV, ADV, and TDF, respectively [15]. In order to characterize this mutant in detail, its replication ability and drug susceptibility were further analyzed (Fig 1).

The results of the Southern blot showed that clone 50–2 exhibited a two-fold higher replication ability than the WT 1.2mer (Fig 1B, left panel). Similar results were obtained from the extracellular viral particles indicating no difference in virion secretion efficiency between WT and clone 50–2 (Fig 1B, right panel). In order to validate the drug resistance of this clone, an *in vitro* drug susceptibility assay was performed using LMV, CLV, ETV, ADV, and TDF. The WT HBV was used as a control; this was discovered to be susceptible to all test drugs (Fig 1C). Clone 50–2 exerted complete resistance to LMV, CLV, and ETV; however, it was susceptible to ADV and TDF (Fig 1D).

These results confirmed clone 50–2 as a multi-drug resistant HBV, with a strong replicative capacity.

### rtL269I substitution enhances replication of WT virus and especially drug-resistant HBV

Since the YMDD mutant M204I is replication defective [10], the 2-fold higher replication ability of the quintuple mutant (harboring the M204I mutation) than the WT led us to investigate the compensatory mutation(s) that rescue DNA replication.

**Fig 2:**
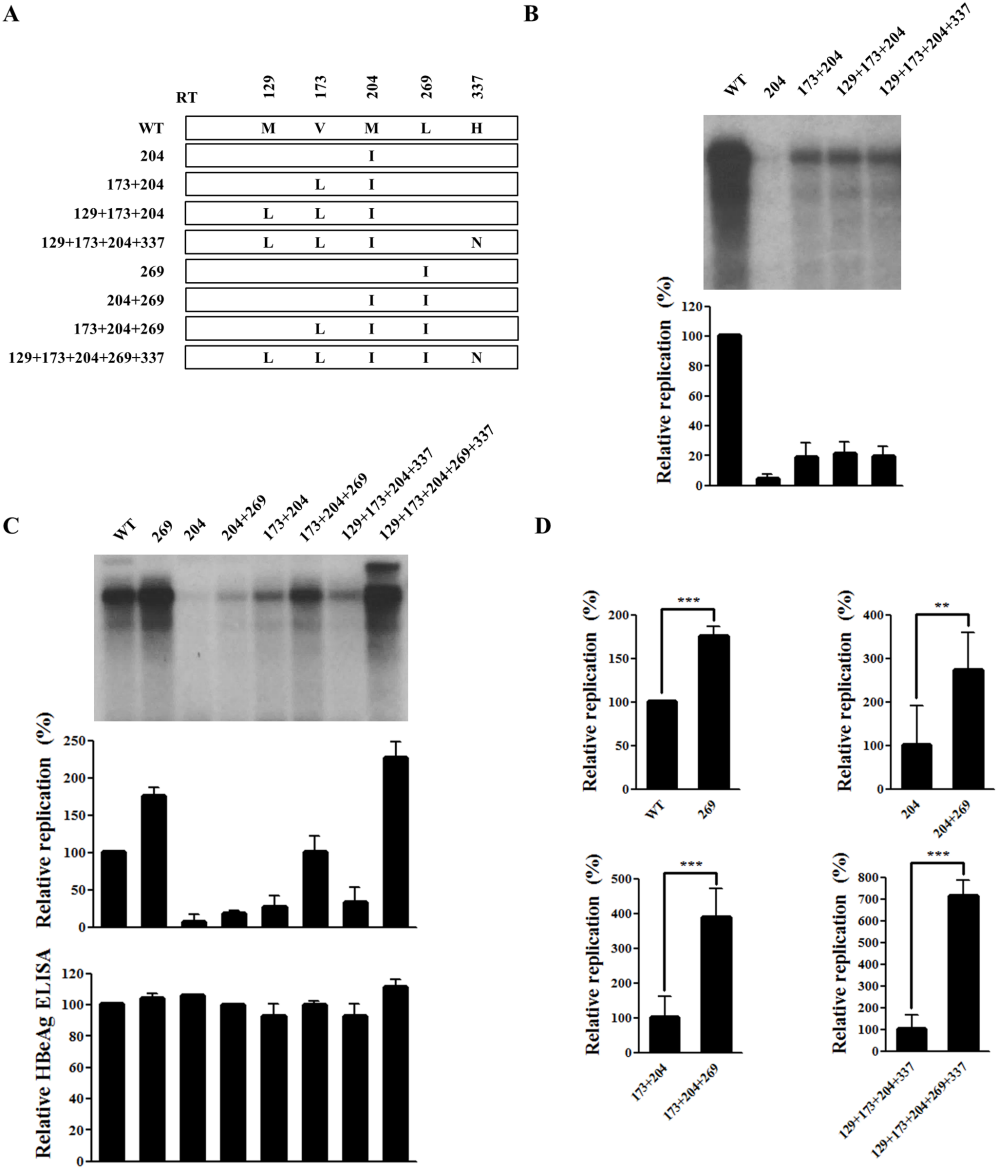
rtL269I substitution enhances the replication of both WT and drug-resistant hepatitis B virus (HBV). (A) Schematic diagram of each HBV mutant construct used in this study. (B–C) Effect of mutations at positions 204, 173, 129, 337, and 269 on HBV DNA replication. Huh7 cells cultured in six-well plates were transfected with HBV plasmids. HBV DNA levels were analyzed by Southern blotting. HBeAg in culture supernatant was determined by ELISA. (D) Phosphor-imager analysis of the relative replication capacities of the HBV mutants. The standard deviation from three independent experiments was calculated (**, *P* < 0.01; ***, *P* < 0.001).

A series of artificial mutants containing part of the quintuple mutation were constructed (Fig 2A). First, the effect of the rtM129L, rtV173L, and rtH337N mutations on the replication of the rtM204I YMDD mutant was tested. Southern blot analysis of four mutants (rtM204I, rtV173L+M204I, rtM129L+V173L+M204I, and rtM129L+V173L+M204I+H337N) was performed to determine the replicative capacity of each clone compared to the WT virus (Fig 2B). As expected, replication of the rtM204I mutant was barely detectable (less than 10% of the WT) [10]. Consistent with previous report [20], rtV173L facilitated replication of the rtM204I mutant. On the other hand, rtM129L and rtH337N did not increase replication of the YMDD mutant (Fig 2B).

Although the rtM129L+V173L+M204I+H337N mutant displayed enhanced replication than the M204I single mutant, the replication efficiency remained less than 10% of the quintuple mutant. Therefore, mutants containing rtL269I (Fig 2A) were constructed, and the effect of rtL269I on the replication of all artificial mutants was determined (Fig 2C).Remarkably, rtL269I substitution resulted in 2- to 7-fold higher replication ability (Fig 2D). The effect of rtL269I was much higher in the mutant backbones and was in the order rtM129L+V173L+M204I+H337N > rtV173L+M204I > rtM204I > WT (approximately 7-fold *vs* 4-fold *vs* 3-fold *vs* 2-fold, respectively). Since the amounts of HBeAg secreted by these mutants were similar (Fig 2C, lower panel) the marked difference in replication level cannot be explained by different transfection efficiencies.

Taken together, our data demonstrated that the substitution at rtL269 is associated with marked rescue of the replication capacity of the multi-drug resistant M204I mutant.

### The rtL269I substitution does not confer drug resistance

**Fig 3:**
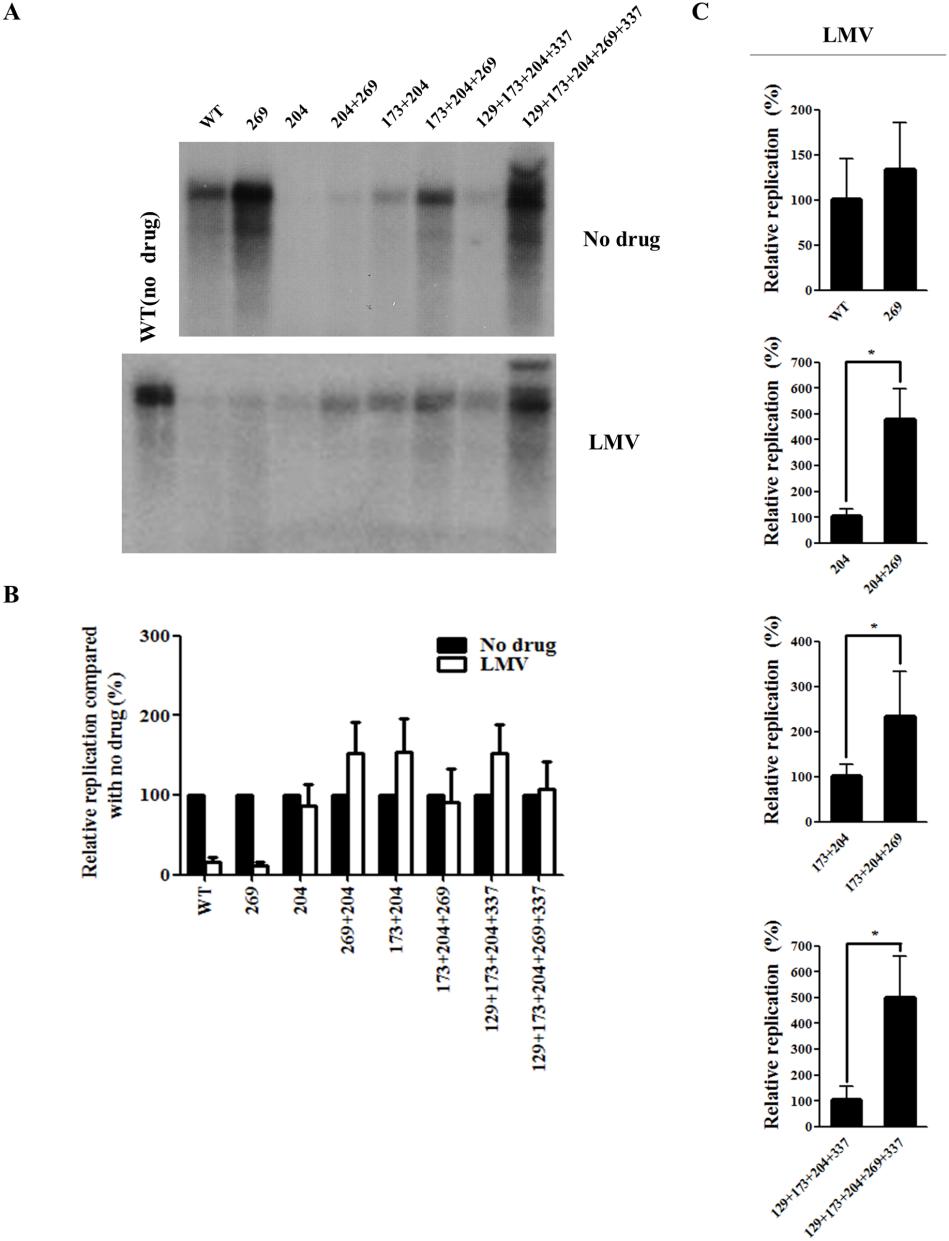
Effect of rtL269I and other substitutions on resistance to lamivudine (LMV). (A) HBV DNA constructs were transfected into Huh7 cells, which were treated with LMV for 3 days. The intracellular HBV DNA was prepared for Southern blot analysis. A representative result has been shown. (B) The relative replication levels of each HBV mutant (no drug *vs* LMV treatment) were calculated based on the results of Figs 2C and 3A. (C) The relative replication ability of the HBV mutants treated with LMV were determined by Southern blotting and quantified by Phosphorimager (*, *P* < 0.05). The relative replication levels of each HBV construct was shown as the mean value of at least three independent experiments.

**Fig 4:**
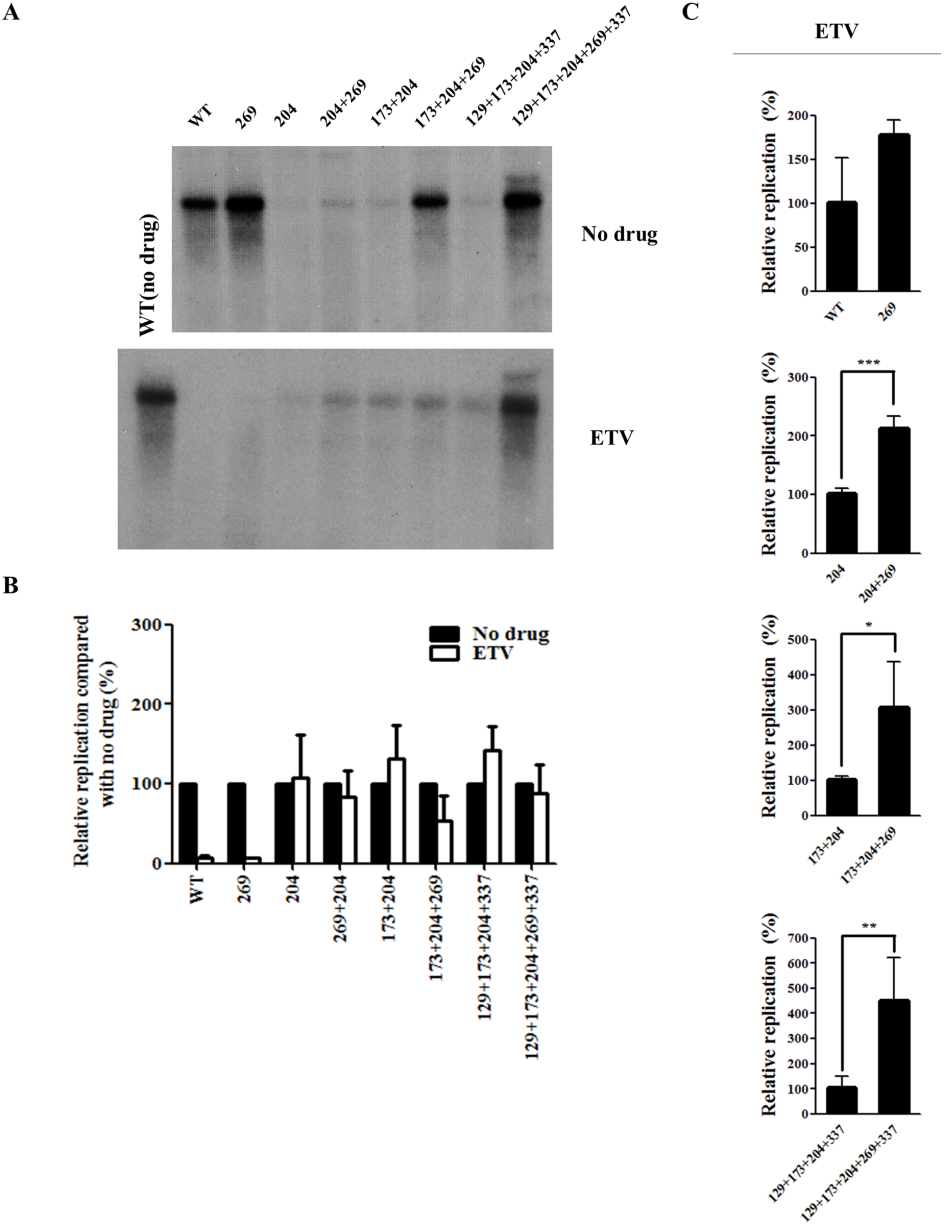
Effect of rtL269I substitution on the resistance to entecavir (ETV). (A) HBV 1.2mer DNA of all the mutants was transfected into Huh7 cells, which were treated with ETV for 3 days. The intracellular HBV DNA level was determined by Southern blot analysis. A representative result was displayed. (B) The relative replication levels of each HBV mutant (no drug *vs* ETV treatment) was calculated based on the results displayed in Figs 2C and 4A. (C) The relative replication ability of the HBV mutants treated with ETV were determined by Southern blot analysis, and quantified by Phosphorimager (*, *P* < 0.05; **, *P* < 0.01; ***, *P* < 0.001). The relative replication level of each HBV construct was displayed as the mean value of at least three independent experiments.

The effect of rtL269I substitution on drug resistance was then investigated. As the quintuple mutant was resistant to LMV and ETV (Fig 1C), the effect of rtL269I on LMV (Fig 3) and ETV (Fig 4) resistance was determined using all artificial mutants.

The rtL269I single mutant was susceptible to LMV and ETV treatment, similar to the WT HBV. When the rtL269I mutation was added to the YMDD mutants, the relative replication levels (no drug *vs* LMV or ETV treatment) of none of the artificial mutants were significantly altered by LMV (Fig 3A and 3B) or ETV treatment (Fig 4A and 4B).

In the presence of LMV (Fig 3C) or ETV (Fig 4C), the rtL269I substitution enhanced replication by approximately 2.5- to 5-fold only when M204I mutation was also present.

Taken together, these results demonstrated that rtL269I is a compensatory mutation for multi-drug resistant YMDD HBV mutants, rather than a drug-resistant mutation.

### The rtL269I substitution may increase polymerase activity through structural change

**Fig 5:**
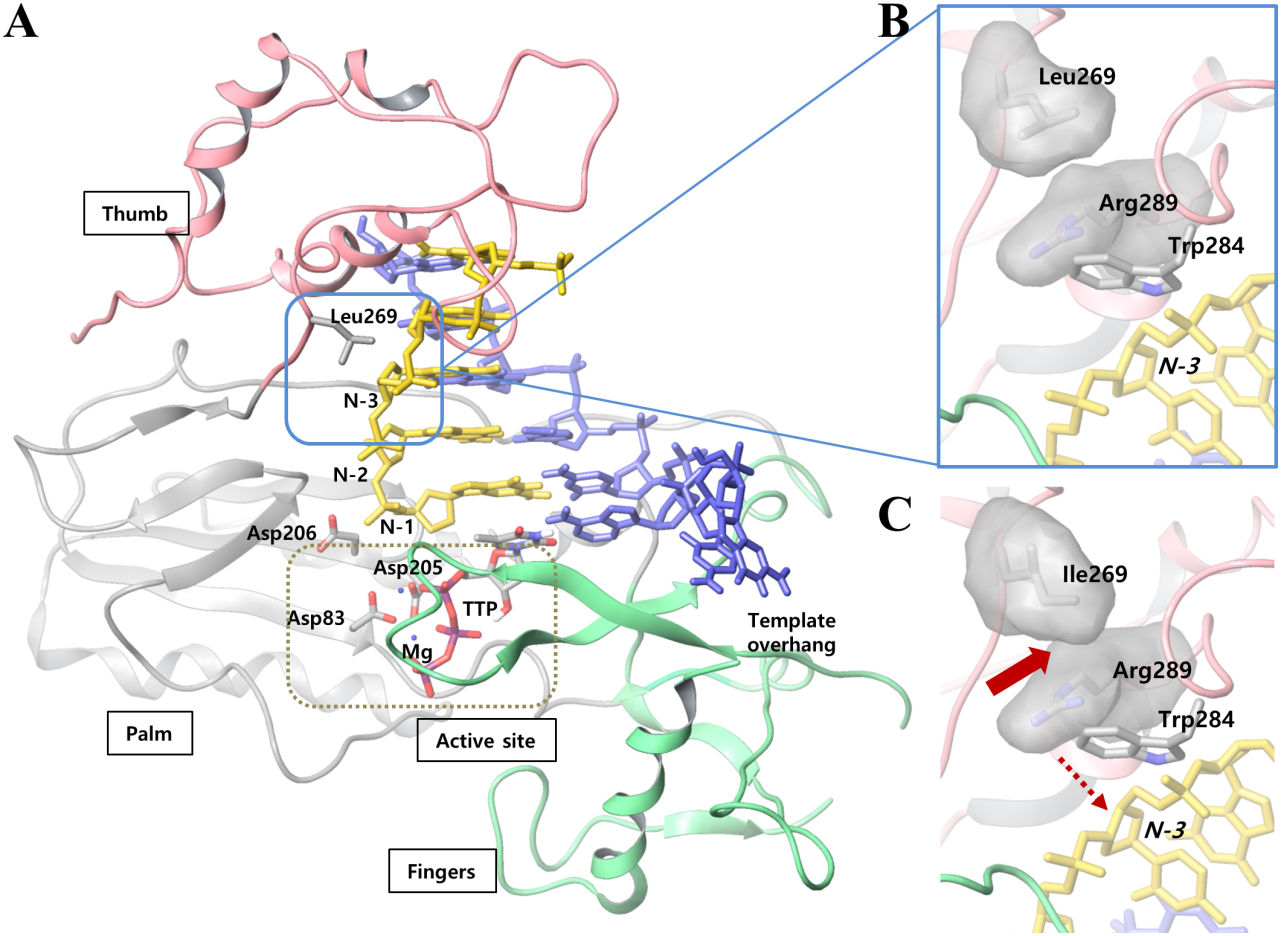
Molecular modeling of rtL269I of HBV polymerase. (A) The three dimensional structure of the modeled HBV polymerase-DNA-TTP complex and (B) the expanded structure around rtL269. N-3 denotes the third nucleotide in the primer strand, starting from the 3′-end, just above the substrate-binding site. The domains of fingers, palm and thumb are shown in green, gray and pink colors, respectively. Also, the primer and template strand are shown in yellow and blue, respectively. TTP and active site residues (Asp83, Asp205 and Asp206) are colored by atom-types. The active site is noted as a dotted box. (C) When Leucine is mutated to isoleucine, residue 269 experiences steric clash with the neighboring rtR289 (block arrow), which would result in a conformational change in the primer binding residue, rtW284, and consequently, in the primer nucleotide, N-3 (dotted arrow).

In order to determine the molecular basis whereby the rtL269I substitution enhances HBV polymerase activity in WT and rtM204I YMDD drug-resistant mutant backbone, a three-dimensional structure of the HBV polymerase was modeled via a comparative modeling study (Fig 5A). rtL269 is positioned far (~27 Å) from the catalytic site of the polymerase; therefore, it can be hardly considered that the substitution with Ile would directly affect the substrate binding event of the catalysis. On the contrary, because of the proximity of rtL269 to the template-binding residue (rtW284), the subtle differences between Leu and Ile might influence the binding affinity of the enzyme to the template nucleotide (*N-3*) (Fig 5B and 5C).

Compared to the original leucine residue, isoleucine at position 269 has a long-chain branch which occupies the same space with the neighboring residue, rtR289 (block arrow in Fig 5C). In order to prevent the resulting steric clash, rtR289 would undergo a conformational change and move away from the bulky side chain of rtI269, which would push the nearby rtW284 closer to the primer strand (dashed arrow in Fig 5C). Thus, our molecular modeling study suggests that the rtL269I substitution causes a conformational change in both RT region and the primer strand. Based on the biochemical results obtained above, it is suggested that the rtL269I substitution locates the primer strand in such a way that the polymerase forms a more efficient ternary complex with the incoming dNTP substrate, which eventually leads to the enhanced activity of rtI269-HBV polymerase in both WT and YMDD mutant.

### Co-emergence of rtL269I substitution and YMDD mutation in CHB patients experiencing treatment failure

The *in vitro* experiments demonstrated that the rtL269I substitution strongly restored HBV polymerase activity of the YMDD mutant. To verify the clinical relevance of this finding, we analyzed HBV DNA sequences from treatment-naïve patients with chronic HBV infection as well as those experiencing sub-optimal response or treatment failure to LMV or CLV.

None of the treatment-naïve patients harbored HBV with the rtL269I substitution (Table 1). Among the four patients treated with CLV, one patient (patient 22) harbored the rtL269I mutation prior to therapy. Of the 22 patients who showed serum HBV DNA content > 60 IU/mL for at least 6 months during LMV or CLV monotherapy. YMDD mutation emerged in 16 patients during the follow-up time of 96 weeks (patients 1 to 18) or 12–17 weeks (patients 19 to 22). Among these 16 patients, 7 (43.8%) displayed the rtL269I substitution (Table 1).

**Table 1:** Mutation profiles in patients exhibiting suboptimal responses during LMV or CLV therapy.

Number	Gender	Age	Genotype	HBeAg	AST (IU/L)	ALT (IU/L)	HBV DNA (IU/mL)	Treatment History	Mutation profiles	rtL269I (prior to therapy)	rtL269I (during therapy)
1	M	47	C2	+	23	15	5.4 × 10^6^	LMV	N/A	-	-
2	F	38	C2	+	17	14	2.4 × 10^3^	LMV	rtL180M, rtM204V, rtL269I	-	+
3	M	45	C2	+	34	39	5.1 × 10^3^	LMV	rtL180M, rtM204V, rtL269I	-	+
4	M	28	C2	+	23	46	4.9 × 10^6^	LMV	rtL180M, rtM204V, rtL269I	-	+
5	M	32	C2	+	21	41	9.1 × 10^6^	LMV→ETV 1.0mg	N/A	-	-
6	M	53	C2	+	27	28	2.4 × 10^4^	LMV	rtM204I	-	-
7	F	54	C2	+	28	24	3.8 × 10^5^	LMV→ETV 1.0mg	N/A	-	-
8	M	55	C2	+	62	78	3.3 × 10^3^	LMV	rtL180M, rtM204V/I	-	-
9	M	28	C2	+	26	46	5.6 × 10^4^	LMV	N/A	-	-
10	F	47	C2	+	22	22	2.4 × 10^2^	LMV	rtL180M, rtM204V	-	-
11	M	46	C2	+	29	48	7.5 × 10^5^	LMV	rtM204I, rtL269I	-	+
12	F	36	C2	+	22	25	5.1 × 10^6^	LMV	rtM204I	-	-
13	F	59	C2	+	58	20	4.2 × 10^6^	LMV→ETV 1.0mg	rtL180M, rtM204V/I, rtL269I	-	+
14	F	46	C2	+	23	7	8.0 × 10^4^	LMV→ETV 1.0mg	N/A	-	-
15	F	41	C2	+	16	18	1.6 × 10^3^	LMV	N/A	-	-
16	F	25	C2	+	28	53	9.8 × 10^4^	LMV	rtL180M, rtM204V	-	-
17	M	47	C2	+	24	20	9.6 × 10^1^	LMV	rtL180M, rtM204V	-	-
18	M	31	C2	+	12	23	1.1 × 10^5^	LMV	rtL180M, rtM204V	-	-
19	F	48	C2	+	34	28	1.1 × 10^7^	CLV	rtM204I	-	-
20	M	39	C2	+	29	24	2.6 × 10^8^	CLV	rtM204I, rtL269I	-	+
21	M	29	C2	+	31	47	5.5 × 10^6^	CLV	rtM204I	-	-
22	F	52	C2	+	35	29	8.0 × 10^5^	CLV	rtM204I, rtL269I	+	+

The so-called “difficult-to-treat” cases, which displayed sustained viral replication despite the complex history of antiviral therapy, were also analyzed. These cases displayed ADV and/or ETV resistance in the milieu of background YMDD mutation, because a sequential antiviral therapy (switch to ADV or ETV 1.0 mg) was generally administered as a rescue therapy in cases of LMV resistance, instead of the combination therapy. This was primarily due to the restricted reimbursement guidelines in Korea at the time of the study. A sequence analysis revealed that almost all patients (13 out of 14 “difficult-to-treat” patients) expressed the rtL269I substitution during the follow-up time (Table 2). Therefore, our clinical data suggest that the rtL269I substitution gradually emerged during treatments, as the genotypic resistance becomes more complex, and that rtL269I is associated with sustained viral replication when subjected to antiviral treatment. Altogether, these results support the *in vivo* relevance of rtL269I substitution as a compensatory mutation in multi-drug resistant HBV.

**Table 2:** Mutation profiles of patients exhibiting “difficult-to-treat” CHB.

Number	Gender	Age	Genotype	HBeAg	AST (IU/L)	ALT (IU/L)	HBV DNA (IU/mL)	Treatment History	Mutation profiles	rtL269I
1	M	51	C2	+	30	42	7.1 x 10^3^	LMV→ADV→ETV 1.0mg→ETV 1.0mg+ADV	rtL180M, rtA181T, rtT184L, rtM204V, rtL269I	+
2	M	52	C2	+	56	123	1.0 x 10^3^	LMV→ADV→ETV 1.0mg→ETV 1.0mg+ADV	rtL180M, rtA181T, rtT184L, rtS202G, rtM204V/I, rtL269I	+
3	M	37	C2	-	45	96	1.8 x 10^5^	LMV→ADV→ETV 1.0mg→ETV 1.0mg+ADV	rtL180M, rtM204V, rtN236T, rtM250V, rtL269I	+
4	M	36	C2	+	85	75	5.9 x 10^5^	LMV→ADV→ETV 1.0mg	rtL180M, rtA181V, rtM204V, rtL269I	+
5	M	58	C2	+	17	15	1.1 x 10^3^	LMV→ADV→ETV 1.0mg→ETV 1.0mg+ADV	rtL180M, rtT184L, rtM204V/I, rtN236T, rtL269I	+
6	M	52	C2	+	38	27	1.9 x 10^5^	LMV→ADV→ETV 1.0mg→ETV 1.0mg+ADV	rtL180M, rtA181V, rtM204V, rtM250V, rtL269I	+
7	M	48	C2	+	36	56	4.2 x 10^5^	LMV→ADV→ETV 1.0mg→ETV 1.0mg+ADV	rtL180M, rtA181T, rt184L, rtM204V, rtN236T, rtL269I	+
8	M	58	C2	+	23	27	6.4 x 10^4^	LMV→ADV→ETV 1.0mg→ETV 1.0mg+ADV	rtL180M, rtA181T, rtM204V, rtN236T, rtM250V, rtL269I	+
9	M	52	C2	+	22	27	1.93 x 10^7^	LMV→LMV+ADV→ETV 1.0mg	rtL180M, rt184L, rtM204V, rtL269I	+
10	M	53	C2	+	42	55	5.20 x 10^2^	ADV→ETV 1.0mg→ETV 1.0mg+ADV	rtL180M, rtT184L/I, rtS202G, rtM204V, rtL269I	+
11	F	67	C2	+	52	26	5.73 x 10^4^	LMV→ETV 1.0mg	rtM204V, rtL269I	+
12*	M	48	C2	+	29	49	8.11 x 10^4^	LMV→ETV 1.0mg	rtL180M, rtM204V	-
13	F	60	C2	+	81	101	5.54 x 10^5^	LMV→ETV 1.0mg→ETV 1.0mg+ADV	rtL180M, rtT184L, rtM204V, rtL269I	+
14	M	45	C2	+	18	22	1.78 x 10^6^	LMV→ETV 1.0mg	rtL180M, rtS202G, rtM204V, rtL269I	+

*Among the 14 “difficult-to-treat” CHB cases, only one did not express an rtL269I mutation

## Discussion

rtM204I/V in the YMDD motif is the primary mutation conferring resistance against LMV, CLV, and ETV. This mutant is almost replication-defective [10]. Therefore, the emergence of compensatory mutations to restore replication capacity is associated with treatment failure and virological breakthrough during antiviral therapies. The compensatory mutations discovered thus far in the YMDD mutants include rtL180M, L80I/V, V173L, S117F, and L229F/V [2, 7, 11–13, 15]. We have previously identified a multi-drug resistant HBV mutant containing the quintuple mutation (rtM129L+V173L+M204I+L269I+H337N), which exhibited robust replication and strong cross-resistance to LMV, CLV, and ETV [15]. In this study, we discovered that the rtL269I substitution is associated with efficient replication in the multi-drug resistant HBV, and thus represents a novel compensatory mutation for the YMDD mutants. We further investigated the mode of action of rtL269I using molecular modeling, and also validated the clinical relevance of this mutation in patients within the “difficult-to-treat” category. Our results suggested that sequence information on the rtL269I substitution would be helpful for the development of treatment regimens for patients demonstrating a sub-optimal response or treatment failure during antiviral therapy.

Molecular modeling is a useful tool to conduct virtual screening and for inhibitor design [21]. The mechanistic characterizations of ETV and ADV binding to the HBV polymerase have been successfully elucidated by molecular modeling [22, 23]. In our molecular modeling strategy, the rtL269I substitution may induce the conformational changes of the template strand, which could enhance replication in the WT and YMDD mutants. Here, it must be noted that the antiviral resistance and reduced polymerase activity conferred by the rtM204I mutant HBV polymerase could be explained by a deformed catalytic site [19]; the bulky side chain of rtM204I protrudes into the active site to form a steric gate, which restricts the binding of the ligand. Therefore, the restoration of the volume of the substrate-binding site by template strand repositioning resulting from rtL269I substitution might be a mechanism counteracting the rtM204I mutation.

It must also be noted that most of the patients (95.5%, 21 out of 22) treated with LMV or CLV monotherapy (primary treatment) did not express the rtL269I substitution before therapy (Table 1). However, among the 16 patients in whom the YMDD mutation was detectable during antiviral treatment, 7 (43.8%) displayed the rtL269I substitution along with rtM204I/V mutation (Table 1). These clinical data suggested that rtM204I/V+L269I was selected during antiviral therapy to confer viral fitness. Because of the retrospective nature of data shown in Table 2, information regarding the HBV mutation profile prior to therapy could not be obtained. Therefore, in order to obtain more conclusive evidence to support our idea, large number of patients with CHB must be recruited for future studies.

In addition, we suggest that the rtL269I substitution emerges as part of complex mutations to maximize viral fitness (Table 2). However, whether the rtL269I substitution can emerge in the milieu of YMDD mutation alone remains to be determined. In fact, in Korea, very few patients express primary resistance to ADV, since an antiviral therapy with ADV as the first-line regimen is not allowed in routine clinical practice. Furthermore, primary resistance to ETV is also rare in treatment-naïve patients subjected to over 5 years of medication [24]. These two limitations necessitate further studies for the elucidation of the possible association between rtL269I mutation and other types of drug-resistant mutations.

Furthermore, as analyzed in Tables 1 and 2, HBV genotype C2 is dominant in Korea. Therefore, we do not know whether the effect of rtL269I substitution is genotype-specific. It will be interesting to further investigate the effect of rtL269I substitution in other HBV genotypes.

Generally, the *in vitro* replication ability of drug-resistant HBV with or without drug treatment is very low. Accordingly, the extremely high replication seen in the quintuple mutant subjected to LMV (Fig 3A) or ETV (Fig 4A) treatment was an unexpected outcome. Although the rtL269I mutation alone could increase the replication capacity (Fig 2D), the rtL269I mutant was susceptible to LMV and ETV, and the mutation did not affect the extent of LMV or ETV resistance (Figs 3B and 4B). When combined with other drug resistant mutations, the effect of rtL269I on the replication ability was found to be backbone-dependent. Approximately 2-, 3-, 4-, and 7-fold higher replication capacity was observed when the rtL269I mutation was introduced to WT, rtM204I, rtV173L+M204I, and rtM129L+V173L+M204I+H337N mutants, respectively (Fig 2D). Similarly, in the presence of antivirals the rtL269I substitution had the maximal effect on replication of the rtM129L+V173L+M204I+H337N mutant (Figs 3C and 4C). The rtM129L+V173L+M204I+H337N mutant showed about 20% of replication level of the WT virus without therapy, whereas the rtM129L+V173L+M204I+L269I+H337N mutant showed 230% replication level (Fig 2C). These data suggested that other mutations cooperate with the rtL269I mutation to achieve maximal viral fitness.

The introduction of several oral antiviral agents for the treatment of CHB has been successful over the past few decades. However, the effects of these agents are threatened by the emergence of primary and compensatory mutations for drug resistance [2]. This is because all the clinically available HBV drugs share a common target (HBV RT). Therefore, in order to ultimately control the HBV, drugs that have targets other than RT must be developed and introduced. In this regard, several trials have been reported for the control of HBV. For this purpose, a heteroaryldihydropyrimidine (HAP) compound has been developed, which targets HBV encapsidation, and demonstrates greater potency than LMV [25]. Glucosidase inhibitors can inhibit HBV replication in the envelope packaging steps in the N-linked glycosylation pathway in *in vivo* targeting [26]. The use of short interfering RNA was successful in the *in vitro* and *in vivo* models [27]. A combination of NA drugs with these non-NA agents could provide an opportunity to overcome the limitations posed by the existing anti-HBV drugs (drug resistance and virological breakthrough).
